# The association between smoking and breast cancer characteristics and outcome

**DOI:** 10.1186/s12885-017-3611-z

**Published:** 2017-09-06

**Authors:** Hadar Goldvaser, Omer Gal, Shulamith Rizel, Daniel Hendler, Victoria Neiman, Tzippy Shochat, Aaron Sulkes, Baruch Brenner, Rinat Yerushalmi

**Affiliations:** 1 Institute of Oncology, Davidoff Cancer Center, Beilinson Hospital, Rabin Medical Center, 39 Jabotinski St., Petach Tikva, Israel; 2 Sackler Faculty of Medicine, Tel Aviv University, POB 39040, Tel Aviv, Israel; 3 Statistical Consulting Unit, Beilinson Hospital, Rabin Medical Center, 39 Jabotinski St., Petach Tikva, Israel

**Keywords:** Breast cancer, Estrogen receptor positive, Smoking, Tobacco

## Abstract

**Background:**

Smoking is associated with an increased incidence of hormone receptor positive breast cancer. Data regarding worse breast cancer outcome in smokers are accumulating. Current literature regarding the impact of smoking on breast cancer characteristics is limited. We evaluated the impact of smoking on breast cancer characteristics and outcome.

**Methods:**

This was a retrospective single center study. All women diagnosed from 4/2005 through 3/2012 and treated in our institute for early, estrogen receptor positive, human epidermal growth factor receptor 2 (HER2) negative breast cancer, whose tumors were sent for Oncotype DX analysis were included. Medical records were reviewed for demographics, clinico-pathological parameters, treatment and outcome. Data regarding smoking were retrieved according to patients’ history at the first visit in the oncology clinic. Patients were grouped and compared according to smoking history (ever smokers vs. never smokers), smoking status (current vs. former and never smokers) and smoking intensity (pack years ≥30 vs. the rest of the cohort). Outcomes were adjusted in multivariate analyses and included age, menopausal status, ethnicity, tumor size, nodal status and grade.

**Results:**

A total of 662 women were included. 28.2% had a history of smoking, 16.6% were current smokers and 11.3% were heavy smokers. Smoking had no impact on tumor size, nodal involvement and Oncotype DX recurrence score. Angiolymphatic and perineural invasion rates were higher in current smokers than in the rest of the cohort (10.4% vs. 5.1%, *p* = 0.045, 8.3% vs. 3.5%, *p* = 0.031, respectively). Smoking had no other impact on histological characteristics. Five-year disease free survival and overall survival rates were 95.7% and 98.5%, respectively. Smoking had no impact on outcomes. Adjusted disease free survival and overall survival did not influence the results.

**Conclusions:**

Smoking had no clinically significant influence on tumor characteristics and outcome among women with estrogen receptor positive, HER2 negative, early breast cancer. As the study was limited to a specific subgroup of the breast cancer population in this heterogeneous disease and since smoking is a modifiable risk factor for the disease, further research is required to clarify the possible impact of smoking on breast cancer.

## Background

There are accumulating data regarding the association between smoking and breast cancer. Mammary tissue is capable to uptake many tobacco carcinogens, including polycyclic aromatic hydrocarbons, aromatic amines and N-nitrosamines. In vitro studies and animal models found that several tobacco carcinogens may induce breast tumors [1, 2] and may cause a more aggressive breast cancer phenotype [3]. Moreover, these carcinogens might cause DNA damage and adduct formation in mammary epithelial cells [4]. Evidence of higher prevalence of these tobacco-related DNA adducts, as well as p53 gene mutations in breast cancer tissue in smokers compared to non-smokers might implicate smoking as a factor in the pathogenesis of breast cancer [5, 6].

The existing literature links smoking with increased breast cancer incidence [7–13]. The updated International Agency for Research on Cancer (IARC) Monographs stated that smoking has a positive association with breast cancer [7]. More specifically, several studies found that smoking was associated with an increased incidence of hormone receptor positive breast cancer incidence, but had no impact on triple negative breast cancer incidence [10, 14].

All-cause mortality rate is higher in smokers with breast cancer compared to non-smokers [15–18]; however, the association of smoking with breast cancer-specific mortality is inconsistent [7]. Several studies found that smoking was associated with worse breast cancer specific survival (BCSS) [19–23], while others did not [24]. Some of the reports that found worse prognosis in patients with breast cancer who smoked were restricted to specific subgroups, such as heavy smokers [25, 26], patients with slow N-acetyltransferase 2 activity or with tumor subtypes other than luminal B [18].

Recent meta-analysis comprising almost 40,000 patients found smoking increases risks of all-cause and breast cancer specific mortality in patients with breast cancer [23].

Smoking is a significant health problem and one of the few potentially modifiable risk factors for breast cancer development. Information regarding the impact of smoking on breast cancer characteristics is scarce. The aim of this study was to evaluate the influence of smoking on breast cancer characteristics and outcome.

We chose to focus on women with early stage, estrogen receptor (ER) positive, human epidermal growth factor receptor 2 (HER2) breast cancer as this group represent the majority of breast cancer patients [27, 28] and there is stronger association between smoking and this subgroup of patients [10, 14]. With the addition of molecular tools, such as the Oncotype-DX (ODX) test to our daily practice [29, 30], patients benefit from an objective prognostic and predictive tool to evaluate risk for disease recurrence. Therefore, only women whose physician ordered the ODX test before deciding on systemic treatment were included in the study.

## Methods

A retrospective single center cohort study was conducted. The study protocol was approved by the Rabin Medical Center Ethics Committee (approval number 0375–14-RMC). All women treated in our institute with ER positive, HER2 negative early breast cancer, who were diagnosed between 4/2005 and 3/2012, and whose tumors were sent for ODX analysis were included. The study cohort was generated using a registry of patients whose tumors were referred to ODX analysis and is kept in our medical center. As data were analyzed anonymously, no consent was given.

The medical records were reviewed up to 8/2015 for demographics, treatment and outcome. Data regarding clinical-pathological parameters were retrieved from patients’ pathological reports. Data regarding history of smoking, number of pack years and smoking status (current vs. former smoker) at breast cancer diagnosis were systematically collected according to the patients’ history during the first visit at the oncology clinic. We defined current smokers as patients who actively smoked at the time of breast cancer diagnosis. Patients with history of smoking were defined as ever smoker.

Patients were grouped and compared according to their history of smoking (ever vs. never smokers), smoking status (current vs. former and never smokers), and smoking intensity (≥30 vs. <30 pack years). Comparisons included tumor size, nodal involvement, stage, ODX recurrence score (RS) and histological characteristics including: ER, progesterone receptor (PR), HER2, grade, angiolymphatic and perineural invasion, Ki67 percent, P53 and histological subtype.

### Immunohistochemical stain reports

ER and PR staining were reported using the modified version of the H-score method: 1 x percentage of weakly staining nuclei +2 x percentage of moderately staining nuclei +3 x percentage of intensely staining nuclei)/100, yielding a range of 0 to 3. Ki67 staining was reported as a percentage of positively stained nuclei (0 to 100%). HER2 negativity was defined as an immunohistochemistry (IHC) test score of 0 or 1. If the IHC score equaled 2, negativity of HER2 was determined according to the fluorescence in situ hybridization test per the American Society of Clinical Oncology guidelines at the test time.

Survival time was defined as time from tissue diagnosis to date of last followup or death. Disease free survival (DFS) was defined as the time between surgery to event (recurrence or death) or end of followup. Duration of followup was determined according to the date of last followup in the chart. Date of recurrence was either the date of biopsy from breast or lymph node (for loco-reginal recurrence) or the date of abnormal imaging (for distant recurrence). Patients’ vital status was ascertained through Israel’s ministry of interior database.

### Statistical analysis

The statistical analysis was generated using SAS Software, Version 9.4. Continuous variables were presented by mean ± SD. Categorical variables were presented by (N, %). T-test was used to compare the value of continuous variables between study groups. Chi-Square (for more than two groups) or Fisher’s exact test (for two groups) were used to compare the value of categorical variables between study groups.

Overall survival (OS) was assessed by Kaplan-Meier survival analysis, with the log-rank test. BCSS and DFS were assessed by the Cox proportional hazards model; with the Fine and Gray correction for competing risks (the competitors were non-cancer death for BCSS and death without recurrence for DFS). Multivariate analysis for OS, BCSS and DFS adjusted for age, menopausal status, ethnicity and tumor characteristics including tumor size, nodal involvement and grade were assessed by the Cox proportional hazards model. Two-sided *p*-values less than 0.05 were considered statistically significant.

## Results

### Patient characteristics

A total of 671 patients with early breast cancer whose tumor was sent to ODX analysis were found. Nine men were excluded, remaining 662 women who were included in the study cohort. Median age was 61 years (range 34–85). 78% women were postmenopausal. Most patients were Ashkenazi Jews (49.4%), followed by Sephardic Jews (41.5%), other ethnicity (7.0%) and Arabs (2.1%).

Data were missing regarding history of smoking in 49 (7.4%) women, smoking status in 52 (7.9%) women and number of pack years in 77 (11.6%) women. History of smoking was present in 173 (28.2%) women, and 101 (16.6%) women were current smokers at the time of breast cancer diagnosis. 66 (11.3%) women were heavy smokers and the median pack years was 23.5 (range 1–140). Patients characteristics and risk factors according to smoking history and status are presented in Table 1. History of smoking and smoking intensity were not associated with age, ethnicity, menopausal status, prior usage of hormone replacement therapy and family history of breast cancer. Current smokers were younger (median age 56 vs. 61, *p* < 0.001), and were more likely to be pre-menopausal (*p* = 0.002, see Table 1). Smoking status had no impact on other characteristics.

**Table 1:** Patients’ characteristics

Population No. patients	All	Hx smoking^a^			Current Smoking^a^			Pack years		
	(662)	Yes (173)	No (439)	*P* value	Yes (101)	No (508)	*P* value	≥30 (66)	0–29 (518)	*P* value
Median Age (range)	61 (34–85)	60 (36–83)	60 (34–85)	0.449	56 (36–75)	61 (34–85)	<0.001	62 (43–83)	60 (34–85)	0.197
Ethnicity										
Ashkenazi	49.5%	46.5%	50.1%	0.165	43.9%	50.0%	0.449	52.3%	49.5%	0.188
Sephardic	41.5%	47.6%	39.1%		49.0%	40.1%		46.2%	40.0%	
Arab	2.1%	0.6%	2.8%		1.0%	2.4%		1.5%	2.2%	
Other	7%	5.3%	8.0%		6.1%	7.5%		0%	8.3%	
Family Hx of breast cancer (yes, %)	39.7%	38%	40.3%	0.608	42.4%	39.3%	0.564	38.5%	40.4%	0.764
Postmenopausal (yes, %)	78%	76.7%	77.4%	0.914	64.4%	79.7%	0.002	85.1%	76.4%	0.122
Hx of HRT usage	21.8%	23.4%	22.2%	0.746	21%	22.8%	0.793	29.2%	21.7%	0.206

^a^Data regarding smoking was retrieved during the first visit at the oncologist clinic. Current smokers were defined as patients who actively smoked at the time of breast cancer diagnosis. Patients with history of smoking were defined as ever smoker

Abbreviations: *HRT* hormonal replacement therapy; Hx-history

- Data were not available for: Ethnicity (*n* = 34); Family history (*n* = 19); HRT usage (*n* = 41); Hx smoking (*n* = 49); Menopausal status (*n* = 28); PY (*n* = 77); smoking status (*n* = 52)

- Cohort included patients with ER positive, HER2 negative disease

History of invasive and non-invasive breast cancer was documented in 11.2% and 2.0% of the women, respectively. Nine percent of the women had a history of malignancy other than breast cancer. Personal history of benign breast disease was noted in 9.1%. Family history (first and second degree) of breast cancer and family history of any other cancer were noted in 39.7% and 58.3% of the women, respectively.

Breast cancer diagnosis was established by screening in the majority of cohort population (83.1%). Screening rate was not associated with history of smoking or active smoking status (*p* = 0.363 and *p* = 0.089, respectively).

### Tumor characteristics

Tumor characteristics are detailed in Tables 2 and 3. Mean tumor size was 1.67 cm (SD 0.78); 83.5% of the women had node negative disease, 7.7% had micrometastatic (0.2–2 mm) nodal involvement, and 8.8% had macroscopic nodal involvement. Presentation was at stage IA in 66.4%, IB in 4.9%, IIA in 23.2%, IIB in 5.2% and IIIA in 0.3%.

**Table 2:** Tumor burden and Oncotype DX recurrence score

Population (num)	Tumor size		Macroscopic node positive^a^		Stage (%)				Oncotype Dx RS	
	Mean cm (SD)	*P*	(%)	*P*	I	II	III	*P*	Mean (SD)	*P*
All (662)	1.67 (0.78)	_	8.8	_	71.3	28.4	0.3	_	19.03 (10.23)	_
Hx smoking^b^										
Yes (173)	1.66 (0.87)	0.642	9.9	0.574	73.8	25.6	0.6	0.695	18.69 (9.15)	0.447
No (439)	1.69 (0.75)		8.5		69.4	30.4	0.2		19.35 (10.78)	
Current smoking^b^										
Yes (101)	1.76 (0.98)	0.392	8	0.73	73.0	26.0	1.0	0.613	19.42 (8.52)	0.70
No (508)	1.67 (0.74)		9.1		70.2	29.6	0.2		19.05 (10.49)	
Number of pack years (PY)										
PY^2^ 0–29 (518)	1.68 (0.76)	0.82	9.5	0.38	69	30.6	0.4	0.172	19.25 (10.66)	0.454
PY > =30 (66)	1.65 (0.96)		6.2		80.0	20.0	0		18.37 (8.68)	

^a^Macroscopic nodes- lymph node metastases >2 mm

^b^Data regarding smoking was retrieved during the first visit at the oncologist clinic. Current smokers were defined as patients who actively smoked at the time of breast cancer diagnosis. Patients with history of smoking were defined as ever smoker

- Abbreviations: *cm* centimeter, *Hx* history, *PY* pack years, *RS* recurrence score, *SD *standard deviation

- Data were not available for: Hx smoking (*n* = 49); nodal status (*n* = 2); PY (*n* = 77); smoking status (*n* = 52); Stage (*n* = 2); Tumor size (*n* = 1)

- Cohort included patients with ER positive, HER2 negative disease

**Table 3:** Histological and molecular characteristics

Population (num)	Histology^a^				Grade		ER		PR		Ki67 (%)		P53 (%)		Angiolymphatic invasion		PNI	
	IDC (%)	ILC (%)	Other (%)	*P*	Mean (SD)	*P*	Mean (SD)	*P*	Mean (SD)	*P*	Mean (SD)	*P*	Mean (SD)	*P*	yes (%)	*P*	yes (%)	*P*
All (662)	80.8	12.4	6.8	-	2.02 (0.59)	-	2.47 (0.57)	-	1.47 (1.19)	-	16 (14)	-	7.0 (17.0)	-	6.0	-	4.4	-
Hx smoking^b^					2		2.47		1.51		16.0		6.0					
smoking^b^	80.3	13.9	5.8		(0.59)		(0.57)		(1.49)		(13.0)		(17.0)		7.3		5.5	
Yes (173)	80.0	12.5	7.5	0.701	2.04	0.535	2.47	0.97	1.48	0.805	16.0	0.854	7.0	0.582	5.4	0.388	3.8	0.358
No (439)					(0.6)		(0.58)		(1.07)		(14.0)		(17.0)					
Current					2.02		2.39		1.4		17.0		7.0					
smoking^b^	81.2	15.8	3.0		(0.58)		(0.59)		(1.08)		(15.0)		(18.0)		10.4		8.3	
Yes (101)	79.7	12.4	7.9	0.16	2.02	0.975	2.49	0.118	1.51		16.0	0.696	7.0	0.879	5.1	0.045	3.5	0.031
No (508)					(0.6)		(0.58)		(1.22)	0.41	(14.0)		(17.0)					
Number of pack years (PY)																		
PY^2^ 0–29					2.03		2.47		1.52		16.0		7.0					
(518)	79.6	12.7	7.7		(0.6)		(0.58)		(1.22)		(14.0)		(17.0)		6.0		4.0	
PY > =30	84.9	12.1	3.0	0.365	2.0	0.712	2.4		1.27		14.0		5.0	0.523	7.7	0.609	7.7	0.18
(66)					(0.53)		(0.62)	0.377	(1.03)	0.112	(12.0)	0.225	(16.0)					

^a^Other subtype histology includes: medullary, mucinous, papillary and tubular carcinoma

^b^Data regarding smoking was retrieved during the first visit at the oncologist clinic. Current smokers were defined as patients who actively smoked at the time of breast cancer diagnosis. Patients with history of smoking were defined as ever smoker

- Abbreviations: *ER* estrogen receptor, *Hx* history, *PNI* perineural invasion, *PR* progesterone receptor, *PY* pack years

- Data were not available for: Angiolymphatic invasion (*n* = 25); Grade (*n* = 116); Hx smoking (*n* = 49); Ki67 (*n* = 179); P53 (*n* = 287); PNI (*n* = 25); PY (*n* = 77); smoking status (*n* = 52)

- Cohort included patients with ER positive, HER2 negative disease

The most common histology was invasive ductal carcinoma (IDC) (80.8%), followed by invasive lobular carcinoma (ILC) (12.4%). Tumors were well, moderately and poorly differentiated in 16.7%, 65.0% and 18.3%, respectively. Perineural and angiolymphatic invasion were noted in 4.4% and 6.0% of the women, respectively. Intensity of IHC staining of ER and PR are detailed in Table 2. As expected, there were correlations between the IHC and the oncotype ER, PR and HER2 according to real time-polymerase chain reaction (RT-PCR) results (*p* < 0.0001 for all variables).

Median ODX score was 17 (range 0–88) with 50.6%, 38.3% and 11.1% of the patients had low (0–17), intermediate (18–30) and high (≥31) RS, respectively.

### Smoking and tumor characteristics

Tumor characteristics according to the evaluated groups are depicted in Tables 2 and 3. Smoking history, smoking status and number of pack years smoked were not associated with tumor and histological characteristics, except with angiolymphatic and perineural invasion, which were higher in current smokers (10.4% vs. 5.1%, *p* = 0.045, 8.3% vs. 3.5%, *p* = 0.031, respectively).

### Treatment

Adjuvant hormonal treatment was administered to 97.7% women, most commonly starting with tamoxifen. Most women (74.4%) did not receive adjuvant chemotherapy. Adjuvant treatment with docetaxel and cyclophosphamide was administered to 11%, anthracycline based chemotherapy without taxane was administered to 9.4%, a combination of anthracycline with cyclophosphamide followed by a taxane to 3.7% and other combinations to 1.5% of the women. History of smoking and active smoking were not associated with adjuvant hormonal or chemotherapy treatment.

### Outcome

Median follow up was 61.9 months (range 1.7–114.6). During this period, 13 (2%) women died of breast cancer, 14 (2.1%) died of other causes and 635 (95.9%) remained alive. Outcome according to smoking groups are depicted in Table 4. Breast cancer recurrence rate was 5.1% for the study cohort. Recurrence rates were 3.6%, 5.3% and 11% for patients with low, intermediate and high RS, respectively (*p* = 0.036). Accordingly, the estimated 5-year DFS rates for all patients were 95.7% and 96.6% for patients with low RS, 95.6% for intermediate RS and 91.8% for high RS. Five-year DFS rate was significantly different between patients with low and high RS (hazard ratio = 0.40, 95% CI 0.16–0.98, *p* = 0.045). History of smoking and smoking status were not associated with recurrence rate (*p* = 0.424 and *p* = 0.128, respectively). DFS rates were not associated with history of smoking (HR = 0.73, 95% CI 0.32–1.68, *p* = 0.456), smoking status (HR = 0.36, 95% CI 0.09–1.48, *p* = 0.155) and number of pack years (HR = 0.85, 95% CI 0.26–2.80, *p* = 0.794).

**Table 4:** Five-year disease free survival, breast cancer specific survival and overall survival

Population (num)^a^	DFS			BCSS			OS		
	5-y DFS	HR 95% CI	*p*	5-y BCSS	HR 95% CI	*p*	5-y OS	HR 95% CI	*p*
All (662)	95.7%	-	-	95.8%	-	-	98.5%	-	-
Hx smoking^b^									
Yes (173)	96.4%	0.73	0.456	99.2%	0.53	0.404	98.8%	0.80	0.74
No (439)	95.1%	(0.32–1.68)		98.4%	(0.12–2.38)		98.6%	(0.22–2.92)	
Current Smokering^b^									
Yes (101)	98.2%	0.36	0.155	NA	NA	NA	NA	NA	NA
No (508)	95.1%	(0.09–1.48)							
Number of pack years (PY)									
PY^2^ 0–29 (518)	95.2%	0.85	0.794	98.5%	0.73		98.6%	1.50	0.596
PY > =30 (66)	96.1%	(0.26–2.80)		99.1%	(0.09–5.70)	0.764	98.5%	(0.33–6.78)	

^a^Sample size: DFS: Smoking history *n* = 599, smoking status *n* = 596, pack years *n* = 575, BCSS: Smoking history *n* = 607, smoking status *n* = 604, pack years *n* = 580, OS: Smoking history *n* = 610, smoking status *n* = 607, pack years *n* = 582

^b^Data regarding smoking was retrieved during the first visit at the oncologist clinic. Current smokers were defined as patients who actively smoked at the time of breast cancer diagnosis. Patients with history of smoking were defined as ever smoker

- Abbreviations: *BCSS* breast cancer specific survival, *DFS* disease free survival, *NA* not applicable, *OS* overall survival

- Cohort included patients with ER positive, HER2 negative disease

Five-year OS rate was 98.5%. OS rates were not associated with history of smoking (HR = 0.80, 95% CI 0.22–2.89, *p* = 0.742), and number of pack years (HR = 1.50, 95% CI 0.33–6.78, *p* = 0.596). Calculation of HR for OS according to smoking status was not applicable. Five-year BCSS was 98.5%. BCSS rates were not associated with smoking status (HR = 0.53, 95% CI 0.12–2.38, *p* = 0.404) and number of pack years (HR = 0.73, 95% CI 0.09–5.70, *p* = 0.764). Calculation of HR for BCSS according to smoking status was not applicable.

Adjustment of HRs for DFS, OS and BCSS for age, menopausal status, ethnicity, tumor size, nodal involvement and grade were similar for history of smoking, smoking status and number of pack years (see Table 5).

**Table 5:** Adjusted Five-year disease free survival, breast cancer specific survival and overall survival^a^

Population^b^ (num)	5-y DFS HR 95% CI	*p*	5-y BCSS HR 95% CI	*p*	5-y OS HR 95% CI	*p*
Hx smoking^c^						
Yes (178)	0.65	0.418	0.66	0.632	1.14	0.852
No (443)	(0.23–1.83)		(0.12–3.58)		(0.28–4.71)	
Current Smoking^3^						
Yes (104)	0.46	0.33	NA	NA	NA	NA
No (513)	(0.10–2.18)					
Number of pack years (PY)						
PY^3^ 0–29 (523)	0.69	0.616	1.02	0.984	2.15	0.344
PY > =30 (67)	(0.16–2.96)		(0.12–8.40)		(0.44–10.49)	

^a^Multivariate adjustment included the following covariates: age, menopausal status, ethnicity, tumor size, nodal involvement and grade

^b^Sample size: DFS: Smoking history *n* = 439, smoking status *n* = 437, pack years *n* = 423, BCSS: Smoking history *n* = 443, smoking status *n* = 441, pack years *n* = 425, OS: Smoking history *n* = 444 smoking status *n* = 442, pack years *n* = 426

^c^Data regarding smoking was retrieved during the first visit at the oncologist clinic. Current smokers were defined as patients who actively smoked at the time of breast cancer diagnosis. Patients with history of smoking were defined as ever smoker

- Abbreviations: *BCSS* breast cancer specific survival, *DFS* disease free survival, *NA* not applicable, *OS* overall survival

- Cohort included patients with ER positive, HER2 negative disease

## Discussion

We evaluated the associations between smoking and ER positive, HER2 negative early breast cancer characteristics and outcome. Current smokers had significantly higher angiolymphatic (*n* = 11) and perineural invasion (*n* = 9) compared to the rest of the study cohort. However, due to the low numbers of patients with these histopathological characteristics, the analysis for this population is limited. Tumor burden, ODX RS and other histological characteristics were not associated with smoking. Lack of differences in traditional prognostic factors such as tumor size, nodal involvement, intensity of IHC stain, grade and Ki67 percent are in line with similar ODX RS in all groups.

Some reports suggested that smoking is associated with increased breast cancer incidence [7–13] and worse outcome [15–23, 31]. However, the magnitude and mechanism of the impact of tobacco on breast cancer development remains unclear, and data regarding the association of smoking and breast cancer characteristics are scarce.

As a result of the decline in smoking prevalence in recent decades observed in many developed countries, former smokers tend to outnumber current smokers [32, 33]. However, in our study the number of current smoker was higher than the number of former smokers (101 vs. 72). This discordance may raise concern for potential bias. A possible explanation could be the inclusion of women whose tumor was sent to ODX analysis. Women who are candidates for adjuvant chemotherapy tend to be younger than the “average” breast cancer population. Indeed, a recent large study evaluating the association between smoking status and breast cancer showed that the number of current smokers might actually outnumber the number of former smokers  [35].

This study did not support previous reports showing worse outcome among patients with breast cancer and history of smoking. Both adjusted and unadjusted analyses of DFS, BCSS and OS showed smoking was not associated with different outcome. However, as only 27 deaths occurred during the followup period, and only 13 deaths were related to breast cancer, conclusions that can be drawn regarding the impact of smoking on outcome in this cohort are limited. Given the excellent outcome for patients with ER positive early breast cancer, a larger population and longer follow-up are required to identify any possible differences.

This study was confined to a specific sub-group of breast cancer patients. In addition, smoking might be associated with molecular and genetic changes which are not expressed by the tumor characteristics collected for this analysis. Therefore, whether smoking has any impact on breast cancer biology remains to be determined.

The study has several limitations. First, as this is a single center, retrospective study, it is vulnerable to unknown bias. Data regarding body mass index (BMI) and physical activity, which have been shown to be associated with breast cancer, were limited and therefore were not adjusted for. Second, smoking history was acquired during the first visit at the oncology clinic and is subjective to recall and reporting biases. Moreover, information regarding smoking status during the followup period was missing, which presents a limitation for outcome analysis according to smoking status. However, we believe the women who were included in this study were unlikely to start smoking given a median age of 61 years. Furthermore, the change in number of heavy smokers during the median followup of 5 years is also probably insignificant. Third, passive smoking, which might also have impact on breast cancer [11, 34], was not recorded. Moreover, age at smoking initiation and history of pack years smoked before first pregnancy were not recorded. Since several reports support the correlation between number of pack years before first pregnancy and breast cancer risk [35, 36], these data might help us understand the influence of smoking on breast cancer better.

Strengths of this study include the large patient cohort. Furthermore, the chart review included detailed, individual patient data, lacking in registry-based studies. The correlation between IHC staining and the ODX ER, PR and HER2 (according to RT-PCR) results, as well as the correlation between ODX RS and the DFS of the study population add to the reliability and validity of the findings.

## Conclusions

In this study cohort, smoking had no clinically significant impact on tumor characteristics and outcome among women with ER positive, HER2 negative early breast cancer. Of note, breast cancer is a heterogeneous disease and these results are limited to a specific subgroup of patients. As smoking is a common health problem and an established modifiable risk factor for breast cancer development, further studies are needed to elucidate the possible impact of smoking on breast cancer patients.

## Abbreviations

BCSS: Breast cancer specific survival
DFS: Disease free survival
ER: Estrogen receptor
HER2: Human epidermal growth factor receptor 2
IDC: Invasive ductal carcinoma
IHC: Immunohistochemistry
ILC: Invasive lobular carcinoma
ODX: Oncotype-DX
OS: Overall survival
PR: Progesterone receptor
RS: Recurrence score
RT-PCR: Real time-polymerase chain reaction
